# The short-, medium-, and long-term prevalence of physical health comorbidities in first-episode psychosis: a systematic review protocol

**DOI:** 10.12688/hrbopenres.13810.1

**Published:** 2023-12-07

**Authors:** Anna Zierotin, Jennifer Murphy, Brian O'Donoghue, Karen O'Connor, Michael Norton, Mary Clarke

**Affiliations:** 1 Department of Psychiatry, University College Dublin, Dublin, Leinster, Ireland; 2 Department of Psychiatry, Royal College of Surgeons in Ireland, Dublin, Leinster, Ireland; 3 St Vincent’s University Hospital, Elm Park, Dublin 4, Ireland; 4 RISE Early Intervention in Psychosis Service, South Lee Mental Health Service, Cork, Ireland; 5 Department of Psychiatry and Neurobehavioural Science, University College Cork, Cork, County Cork, Ireland; 6 Recovery and Engagement Lead, Office of Mental Health Engagement and Recovery, Health Service Executive, County Dublin, Ireland; 7 DETECT Early Intervention for Psychosis Service, County Dublin, Ireland

**Keywords:** First-episode Psychosis, Physical Health Comorbidities, Metabolic Health, Cardiovascular Health, Respiratory Health, Cancer

## Abstract

**Background:**

Individuals with first-episode psychosis (FEP) face an increased risk of physical comorbidities, notably cardiovascular diseases, metabolic disorders, respiratory disorders, and certain types of cancer. Previous reviews report pooled physical health prevalence from chronic psychosis and FEP groups. By contrast, this review will focus on antipsychotic-naïve FEP cohorts and incorporate data from observational longitudinal studies and antipsychotic intervention studies to understand the progression of physical health comorbidities from the onset to later stages of psychosis. This review aims to systematically examine the short-, medium-, and long-term period prevalence of these comorbidities in FEP and variations related to demographic factors.

**Methods:**

A systematic review will be conducted using the PRISMA and MOOSE guidelines. Medline, Embase, PsycINFO, and CINAHL+, as well as Clinical Trials gov.uk, OpenGrey, WHO International Clinical Trials Registry Platform, Current Controlled Trials, United States National Institute of Health Trials Registry, and the Irish Health Repository, will be searched from inception. Longitudinal studies exploring physical health outcomes in FEP cohorts and antipsychotic intervention studies monitoring health outcomes in antipsychotic naïve FEP individuals will be eligible for inclusion. Two reviewers will independently screen titles, abstracts, and full-text articles. Bias in individual studies will be assessed using the JBI Critical Appraisal Checklist. A meta-analysis of the short-, medium-, and long-term prevalence of cardiovascular, metabolic, cancer, and respiratory outcomes and a narrative synthesis will be conducted. If possible, a meta-regression on the impact of demographic variables will be conducted.

**Conclusions:**

This systematic review will clarify the progression of physical health comorbidities in FEP, informing early intervention strategies and policies for this population. Subsequent findings will be submitted to a leading journal, supplemented by a recovery education module for patient groups and a lay summary for wider dissemination.

**Registration:**

The study was registered in PROSPERO, the International Prospective Register of Systematic Reviews ( CRD42023431072; 17/06/2023).

## Introduction

Individuals with psychosis face an increased risk of various physical health conditions, including cardiovascular diseases, respiratory disorders, metabolic disorders, and cancer ( De Hert *et al.*, 2011; Leucht *et al.*, 2007). The increased incidence of physical comorbidities, especially cardiovascular diseases, contributes to a significant burden of physical illness and premature mortality, with individuals with psychosis experiencing a reduced life expectancy of approximately 10–20 years compared to the general population ( Firth *et al.*, 2019). A higher prevalence of lifestyle risk factors such as smoking, poor nutrition, and disrupted sleep patterns in people with psychosis contribute to these health risks ( Firth *et al.*, 2019). Additionally, shared risk factors, including non-white ethnicity and social deprivation, contribute to distinct multimorbidity patterns in psychosis ( Lawrence & Kisely, 2010; Rodrigues *et al.*, 2021). Recent research has also reported the genetic risk for schizophrenia to be associated with cardiac structural changes that can worsen cardiac outcomes ( Pillinger *et al.*, 2023). This connection further emphasises the complex interplay between genetic and lifestyle factors in shaping the health outcomes of those with psychosis.

Metabolic syndrome, encompassing dyslipidaemia, hypertension, impaired glucose regulation, and central obesity, is prevalent in up to 69% of those with chronic schizophrenia ( Vancampfort *et al.*, 2015). Although antipsychotic use may contribute to these metabolic changes, altered glucose homeostasis is already observed in antipsychotic-naïve individuals with first-episode psychosis (FEP), suggesting that metabolic disturbances emerge from the onset of psychosis ( Pillinger *et al.*, 2017a; Pillinger *et al.*, 2017b).

Research has indicated that two-thirds of individuals with FEP will experience metabolic changes, such as impaired glucose and lipid metabolism, as well as weight gain exceeding 7% during the initial 12 months of treatment ( Alvarez-Jiménez *et al.*, 2008; Coentre *et al.*, 2022). These metabolic changes lead to a greater risk of cardiovascular disease, stroke, and type 2 diabetes later in life, which underlines the importance of early intervention and the need for attention to physical health changes from the initial diagnosis of psychosis. Moreover, individuals with schizophrenia face a higher risk of respiratory conditions such as chronic obstructive pulmonary disease (COPD) and pneumonia ( Suetani *et al.*, 2021), which might be linked to increased active and passive smoke exposure ( Hunter *et al.*, 2020; Lally *et al.*, 2019), childhood exposure to air pollution ( Antonsen *et al.*, 2020; Schraufnagel *et al.*, 2019) and socioeconomic factors ( Rocha *et al.*, 2020; Sariaslan *et al.*, 2016).

Research on the incidence of cancer in people with psychosis has been mixed, with earlier studies concluding a generally lower cancer incidence in this population ( Ananth & Burnstein, 1977) and more recent reviews finding an increased risk of certain cancer types but a decrease for others ( Wootten *et al.*, 2022; Xu *et al.*, 2017; Zhuo & Triplett, 2018). The inconsistencies between earlier and more recent studies may partially reflect the evolving practices in diagnosing and reporting cancer among those with psychosis, emphasising the importance of updating previous findings with more contemporary health data.

Despite several systematic reviews establishing the increased prevalence of physical health disorders in individuals with psychosis ( Leucht *et al.*, 2007; Oud & Meyboom-de Jong, 2009; Suetani *et al.*, 2021; Vancampfort *et al.*, 2013), these reviews often report pooled prevalence data from individuals with chronic psychosis and FEP, or only report FEP baseline outcomes, limiting our understanding of the evolution of physical health outcomes in FEP. Therefore, by focusing specifically on FEP cohorts, this review aims to understand the progression of physical health comorbidities from the onset to the later stages of psychosis.

Given the limited number of longitudinal FEP cohort studies reporting physical health outcomes ( Suetani *et al.*, 2021; Vancampfort *et al.*, 2013; Wootten *et al.*, 2022), this review will also synthesise evidence from antipsychotic intervention studies. Antipsychotic intervention studies often conduct baseline and follow-up physical health monitoring, providing data on health outcomes, particularly metabolic health. Given that antipsychotic medication commonly constitutes first-line treatment in FEP, including these studies will allow us to capture more data about the development of physical health outcomes. While we acknowledge that clinical trials may not generalise to the broader FEP population due to specific inclusion and exclusion criteria, we aim to analyse outcomes separately and then provide a comprehensive pooled analysis.

### Review questions

The primary objective of this review is to systematically examine the existing evidence relating to the short-, medium- and long-term period prevalence of cardiovascular, metabolic, cancer and respiratory comorbidities in individuals with first-episode psychosis.

1. What is the short-, medium- and long-term period prevalence of cardiovascular, metabolic, cancer and respiratory comorbidities in individuals with first-episode psychosis?

2. Does the prevalence of cardiovascular, metabolic, cancer and respiratory comorbidities vary with certain demographic factors such as age, sex, or ethnicity among individuals with first-episode psychosis?

## Methods

### Study registration

The study was registered in PROSPERO, the International Prospective Register of Systematic Reviews ( CRD42023431072; 17/06/2023). This protocol follows the PRISMA-P (Preferred Reporting Items for Systematic Reviews and Meta-Analysis Protocols; Moher *et al.*, 2015; Zierotin *et al.*, 2023) guidelines. The review will be conducted according to the Preferred Reporting Items for Systematic Review and Meta-Analyses (PRISMA) 2020 statement ( Page *et al.*, 2021) and the Meta-analyses of Observational Studies in Epidemiology (MOOSE) guidelines ( Stroup *et al.*, 2000).

### Eligibility criteria

This review will include research reporting on the relationship between FEP and cardiovascular, metabolic, cancer and respiratory health outcomes. This will include two study types: 1) observational longitudinal studies investigating physical health outcomes in a FEP cohort and 2) antipsychotic intervention studies reporting the incidence of physical health outcomes in antipsychotic-naïve individuals with FEP. We will exclude single case studies and case series. We will also exclude studies that did not measure physical health outcomes or only measured physical health at baseline. There will be no restrictions regarding language or publication period. Studies will be selected for inclusion in the systematic review according to the following population and outcome criteria.

### Population

The population includes individuals with a first-episode diagnosis of psychosis, either clinician-diagnosed, confirmed through medical records, or enrolled in a clinical program dedicated to psychosis (for example, Early Intervention in Psychosis). First-episode psychosis is defined as first treatment contact (inpatient or outpatient) regardless of the duration of untreated illness. There are no restrictions regarding the age of participants. For studies to be eligible for inclusion, individuals with FEP should have no more than 28 days of antipsychotic drug exposure at the minimal effective dose, as recommended by the Maudsley clinical guidelines ( Owen *et al.*, 2014). However, if a study’s cohort contains less than 5% of individuals with antipsychotic drug exposure longer than 28 days, it will still be considered for inclusion. Studies solely investigating organic psychotic disorders or personality disorders such as schizoid or schizotypal personality disorder will be excluded. Moreover, research focusing on individuals with psychosis with comorbid neurodivergence or neurodevelopmental disorders will also be excluded. However, if a study’s cohort contains less than 20% of individuals with the conditions above, it will still be considered for inclusion. If mixed samples (for example, severe mental illness groups) are used, we will attempt to extract the data specific to people with psychosis. If data extraction is not possible, we will contact the authors twice within a one-month period to request the data for individuals with psychosis. If multiple reports of the same study are identified, the most complete report will be included.

### Outcomes

The primary outcome is the prevalence or incidence of metabolic, cardiovascular, cancer or respiratory outcomes in individuals with FEP. Physical health outcomes must be confirmed by a clinician, enrolment in a clinical program dedicated to the physical illness, medical records, or primarily according to ICD-10 criteria. However, for interpretation consistency and considering recent updates, any ambiguities in outcomes will be assessed against the ICD-11 criteria. Eligible studies must report the prevalence/incidence of these health outcomes at a specified follow-up time point post the initial psychosis diagnosis, meaning the studies should specify the duration from the first diagnosis of psychosis to the time when these physical health outcomes were reported. There are no restrictions regarding the study time frame. In the context of this review, the terms “short-”, “medium-” and “long-term prevalence” refer to the occurrence of cardiovascular, metabolic, cancer, and respiratory comorbidities in individuals diagnosed with first-episode psychosis (FEP) over specific time intervals post-diagnosis. Specifically, “short-term prevalence” assesses these comorbidities from >0–12 months following an FEP diagnosis, “medium-term prevalence” spans from 13–36 months post-diagnosis, and “long-term prevalence” encompasses both 37–60 months post-diagnosis and periods exceeding 5 years. The secondary outcome of interest concerns the impact of demographic and study variables, such as age, sex, ethnicity and study year, on the prevalence of cardiovascular, metabolic, cancer and respiratory comorbidities.

### Information sources

The following databases will be searched from inception for publications: Medline, Embase, PsycInfo, and CINAHL+. Grey literature, including ClinicalTrials gov.uk, OpenGrey, WHO International Clinical Trials Registry Platform, Current Controlled Trials, United States National Institute of Health Trials Registry, and the Irish Health Repository, will be searched. The searches will be re-run prior to the final analysis.

### Search strategy

The literature search strategy will use Medical Subject Headings (MeSH) and text words related to first-episode psychosis, metabolic, cardiovascular, cancer and respiratory outcomes. The search terms for the physical disorders are adapted from previous publications on similar topics ( Leucht *et al.*, 2007; Onyeka *et al.*, 2019). The search strategy was produced with the help of a librarian and follows the below structure:

First-episode psychosis terms AND metabolic outcomes OR cardiovascular outcomes OR cancer terms OR respiratory outcomes

The full search strategy can be found in Table 1.

**Table 1.** This table lists the search terms, and Boolean operators used to identify studies on psychotic disorders and their associations with cardiovascular, metabolic, respiratory and cancer outcomes. The keywords are grouped by database (Medline, PsycINFO, EMBASE, CINAHL+) to illustrate the tailored search strategies used for each database.

Concept	Database				Keywords
	Medline (via Ovid)	PsycNFO (via Ovid	EMBASE (Via Ovid)	CINAHL+ (via Ebesco)	
Psychotic disorders AND	((first episode* or first-episode* or first* or recent or early* or new* or initial* or "newly admitted") adj2 (psychos?s or psychotic or schizo* or delusion* or paranoi*))	((first episode* or first-episode* or first* or recent or early* or new* or initial* or "newly admitted") adj2 (psychos?s or psychotic or schizo* or delusion* or paranoi*))	((first episode* or first-episode* or first* or recent or early* or new* or initial* or "newly admitted") adj2 (psychos?s or psychotic or schizo* or delusion* or paranoi*))	((“first episode*” or “first-episode*” or first* or recent or early* or new* or initial* or “newly admitted”) N2 (psychos?s or psychotic or schizo* or delusion* or paranoi*))	First-episode psychosis or first-episode schizophrenia/delusional disorder/paranoid disorder
Cancer OR	(Cancer* or neoplasm* or carcinoma* or carcinogen* or (malignan* adj (tumo?r* or lesion)) or metastas* or melanoma? or sarcoma? or Mesothel?oma or polycyth?emia vera or Myelodysplastic or leukemia or leucaemia or myelofibrosis or cyst? or neurofibroma? or glioma? or lymphoma?) or exp neoplasms/	(Cancer* or neoplasm* or carcinoma* or carcinogen* or (malignan* adj (tumo?r* or lesion)) or metastas* or melanoma? or sarcoma? or Mesothel?oma or polycyth?emia vera or Myelodysplastic or leukemia or leucaemia or myelofibrosis or cyst? or neurofibroma? or glioma? or lymphoma?) or exp neoplasms/	(Cancer* or neoplasm* or carcinoma* or carcinogen* or (malignan* adj (tumo?r* or lesion)) or metastas* or melanoma? or sarcoma? or Mesothel?oma or polycyth?emia vera or Myelodysplastic or leukemia or leucaemia or myelofibrosis or cyst? or neurofibroma? or glioma? or lymphoma?) or exp neoplasm/	Cancer* or neoplasm* or carcinoma* or carcinogen* or (malignan* W1 (tumo#r* or lesion)) or metastas* or melanoma# or sarcoma# or Mesothel#oma or polycyth#emia vera or Myelodysplastic or leukemia or leucaemia or myelofibrosis or cyst# or neurofibroma# or glioma# or lymphoma# or (MH "Neoplasms+")	Cancer or neoplasms or malignant tumour or malignant lesion or carcinoma or melanoma or metastasis or sarcoma or mesothelioma or polycythemia vera or leukaemia or myelodysplastic syndromes or myelofibrosis or neurofibroma or glioma or lymphoma or cyst
Metabolic outcomes	(Diabet* or T2DM or mellitus or glucose or hyperglycaemia or insulin or prediabet* or cholesterol or triglycerides or "blood pressure" or metabolic or hypertensi* or hyperlipid* or hyperlipo* or lipoprotein* or MetS or HbA1c or cardiometabolic or "waist circumference" or "body mass index" or BMI or hypercholesterol* or hypertriglycerid* or "high density lipoprotein" or "low density lipoprotein") or exp diabetes mellitus/ or exp metabolic syndrome X/ or exp dyslipidemia/ or exp hypertension/ or exp cholesterol/ or exp hyperlipoproteinemia/ or exp hypertriglyceridemia/ or exp hyperlipidemia/ or exp Hypercholesterolemia/ or exp obesity/ or exp Hyperglycemia/ or exp obesity/ or exp cholesterol/ or exp insulin/	(Diabet* or T2DM or mellitus or glucose or hyperglycaemia or insulin or prediabet* or cholesterol or triglycerides or "blood pressure" or metabolic or hypertensi* or hyperlipid* or hyperlipo* or lipoprotein* or MetS or HbA1c or cardiometabolic or "waist circumference" or "body mass index" or BMI or hypercholesterol* or hypertriglycerid* or "high density lipoprotein" or "low density lipoprotein") or exp diabetes mellitus/ or exp metabolic syndrome X/ or exp hypertension/ or exp cholesterol/ or exp hyperlipoproteinemia/ or exp hypertriglyceridemia/ or exp hyperlipidemia/ or exp Hypercholesterolemia/ or exp obesity/ or exp Hyperglycemia/ or exp obesity/ or exp cholesterol/ or exp insulin/	(Diabet* or T2DM or mellitus or glucose or hyperglycaemia or insulin or prediabet* or cholesterol or triglycerides or "blood pressure" or metabolic or hypertensi* or hyperlipid* or hyperlipo* or lipoprotein* or MetS or HbA1c or cardiometabolic or "waist circumference" or "body mass index" or BMI or hypercholesterol* or hypertriglycerid* or "high density lipoprotein" or "low density lipoprotein") or exp diabetes mellitus/ OR exp metabolic syndrome X/ OR exp dyslipidemia OR exp hypertension/ OR exp cholesterol/ OR exp hyperlipoproteinemia/ OR exp hypertriglyceridemia/ OR exp hyperlipidemia OR exp Hypercholesterolemia/ OR exp obesity/ OR exp Hyperglycemia/ OR exp obesity/ OR exp cholesterol/ OR exp insulin	(Diabet* or T2DM or mellitus or glucose or hyperglycaemia or insulin or prediabet* or cholesterol or triglycerides or "blood pressure" or metabolic or hypertensi* or hyperlipid* or hyperlipo* or lipoprotein* or MetS or HbA1c or cardiometabolic or "waist circumference" or "body mass index" or BMI or hypercholesterol* or hypertriglycerid* or "high density lipoprotein" or "low density lipoprotein") or (MH "Metabolic Syndrome X+") OR (MH "Hypertension+") OR (MH "Obesity+") OR (MH "Cholesterol+") OR (MH "Hyperglycemia+") OR (MM "Diabetes Mellitus, Type 2") OR (MH "Hypercholesterolemia+") OR (MH "Hyperlipoproteinemia+") OR (MH "Insulin Resistance+")	Diabetes mellitus or glucose or insulin or hyperglycaemia or prediabetes cholesterol or triglycerides or blood pressure or metabolic syndrome or hypertension or hyperlipidaemia or lipoprotein or HbA1c or cardiometabolic or waist circumference or body mass index or BMI or hypercholesterolemia or hypertriglyceridemia or high density lipoprotein or low density lipoprotein
Respiratory outcomes OR	(((respiratory or lung) adj (disease or condition or infection* or inflammation or disorder* or aspiration or syndrome)) or asthma or bronch?t?s or emphysema or pulmon?ry or COPD or pneumon* or tuberculosis or apn?ea or apneia or airway obstruct* or chronic obstruct* or infl?enza or sinusitis or pharyngitis or tonsillitis or laryng?t?s or dyspnoea or pleuritis or pl?urisy or orthopnoea or nasal polyps or rhinitis or cough or tracheitis or empyema or bronchial or SARS or asphyx?a or D?spha?ia or tuberculosis) or *Respiratory Tract Diseases/ or *Bronchial Diseases/ or *Asthma/ or *Bronchitis/ or *Laryngeal Diseases/ or *Lung Diseases/ or *Nasal Obstruction/ or *Nasal Polyps/ or *Sinusitis/ or *Rhinitis/ or *Pleural Diseases/ or *Pleurisy/ or *Tuberculosis, Pleural/ or *Apnea/ or *Cough/ or *Dyspnea/ or *Respiratory Aspiration/ or *Airway Obstruction/ or *Common Cold/ or *Empyema, Pleural/ or *Influenza, Human/ or *Laryngitis/ or *Pharyngitis/ or *Pleurisy/ or *Pneumonia/ or *Rhinitis/ or *Severe Acute Respiratory Syndrome/ or *Sinusitis/ or *Tracheitis/ or *Tuberculosis/	(((respiratory or lung) adj (disease or condition or infection* or inflammation or disorder* or aspiration or syndrome)) or asthma or bronch?t?s or emphysema or pulmon?ry or COPD or pneumon* or tuberculosis or apn?ea or apneia or airway obstruct* or chronic obstruct* or infl?enza or sinusitis or pharyngitis or tonsillitis or laryng?t?s or dyspnoea or pleuritis or pl?urisy or orthopnoea or nasal polyps or rhinitis or cough or tracheitis or empyema or bronchial or SARS or asphyx?a or D?spha?ia or tuberculosis) or *Respiratory Tract Diseases/ or *Bronchial Diseases/ or *Asthma/ or *Bronchitis/ or *Laryngeal Diseases/ or *Lung Diseases/ or *Nasal Obstruction/ or *Nasal Polyps/ or *Sinusitis/ or *Rhinitis/ or *Pleural Diseases/ or *Pleurisy/ or *Tuberculosis, Pleural/ or *Apnea/ or *Cough/ or *Dyspnea/ or *Respiratory Aspiration/ or *Airway Obstruction/ or *Common Cold/ or *Empyema, Pleural/ or *Influenza, Human/ or *Laryngitis/ or *Pharyngitis/ or *Pleurisy/ or *Pneumonia/ or *Rhinitis/ or *Severe Acute Respiratory Syndrome/ or *Sinusitis/ or *Tracheitis/ or *Tuberculosis/	(((respiratory or lung) adj (disease or condition or infection* or inflammation or disorder* or aspiration or syndrome)) or asthma or bronch?t?s or emphysema or pulmon?ry or COPD or pneumon* or tuberculosis or apn?ea or apneia or airway obstruct* or chronic obstruct* or infl?enza or sinusitis or pharyngitis or tonsillitis or laryng?t?s or dyspnoea or pleuritis or pl?urisy or orthopnoea or nasal polyps or rhinitis or cough or tracheitis or empyema or bronchial or SARS or asphyx?a or D?spha?ia or tuberculosis) or *Respiratory Tract Diseases/ or *Bronchial Diseases/ or *Asthma/ or *Bronchitis/ or *Laryngeal Diseases/ or *Lung Diseases/ or *Nasal Obstruction/ or *Nasal Polyps/ or *Sinusitis/ or *Rhinitis/ or *Pleural Diseases/ or *Pleurisy/ or *Tuberculosis, Pleural/ or *Apnea/ or *Cough/ or *Dyspnea/ or *Respiratory Aspiration/ or *Airway Obstruction/ or *Common Cold/ or *Empyema, Pleural/ or *Influenza, Human/ or *Laryngitis/ or *Pharyngitis/ or *Pleurisy/ or *Pneumonia/ or *Rhinitis/ or *Severe Acute Respiratory Syndrome/ or *Sinusitis/ or *Tracheitis/ or *Tuberculosis/	(((respiratory or lung) adj (disease or condition or infection* or inflammation or disorder* or aspiration or syndrome)) or asthma or bronch#t#s or emphysema or pulmon#ry or COPD or pneumon* or tuberculosis or apn#ea or apneia or airway obstruct* or chronic obstruct* or infl#enza or sinusitis or pharyngitis or tonsillitis or laryng#t#s or dyspnoea or pleuritis or pl#urisy or orthopnoea or nasal polyps or rhinitis or cough or tracheitis or empyema or bronchial or SARS or asphyx#a or D#spha#ia or tuberculosis) or (MM "Asthma") OR (MM "Bronchitis") OR (MM "Laryngitis") OR (MM "Laryngeal Diseases") OR (MM "Lung Diseases") OR (MM "Pneumonia") OR (MM "Apnea") OR (MM "Cough") OR (MM "Aspiration") OR (MM "Dyspnea") OR (MH "Rhinitis, Allergic, Perennial") OR (MH "Rhinitis, Allergic, Seasonal") OR (MM "Common Cold") OR (MM "Empyema") OR (MM "Influenza") OR (MM "Pharyngitis") OR (MM "Pleurisy") OR (MM "Rhinitis") OR (MM "Severe Acute Respiratory Syndrome") OR (MM "Sinusitis") OR (MM "Tonsillitis") OR (MM "Tuberculosis, Pulmonary")	Respiratory disease or lung disease or asthma or bronchitis or emphysema or pulmonary disease or COPD or pneumonia or tuberculosis or apnoea or influenza or airway obstructive or sinusitis or influenza or pharyngitis or tonsillitis or laryngitis or pharyngitis or pleuritis or dyspnoea or pleuritis or orthopnoea or empyema or cough or tracheitis nasal polyp or tuberculosis or SARS or asphyxia or dysphagia or tuberculosis
Cardiovascular outcomes	(cardiovascular or Cardiovascular disease* or ((coronary or isch#emic) adj2 (disease or occlusion or stenos#s or thrombos#s)) or (myocardial adj (isch#emia or infarct*)) or ((coronary or myocardial or heart or cardiac) adj2 (revasculari#ation or angioplasty or atherectomy or bypass)) or ((heart or cardiac or ventricular) adj failure) or angina or ((ventricular or systolic or diastolic) adj (dysfunction or impairment)) or (stroke or cerebrovascular accident) or ((brain or cerebral or intracranial) adj2 (infarct* or thrombos?s or embolism)) or transient isc#emic attack or tachycardi* or heart attack* or (heart adj2 (attack or disease)) or left ventricular hypertrophy) or Cardiovascular Diseases/ or exp Myocardial Ischemia/ or exp Angina Pectoris/ or exp Myocardial Revascularization/ or exp Heart Failure/ or exp Ventricular Dysfunction/ or exp brain ischemia/ or exp intracranial arterial diseases/ or exp "intracranial embolism and thrombosis"/ or exp stroke/	“cardiovascular disease*” OR ((coronary or isch?emic) adj2 (disease or occlusion or stenos#s or thrombos#s)) OR (myocardial adj (isch?emia or infarct*)) OR ((coronary or myocardial or heart or cardiac) adj2 (revasculari?ation or angioplasty or atherectomy or bypass)) OR ((heart or cardiac or ventricular) adj failure) OR angina OR ((ventricular or systolic or diastolic) adj (dysfunction or impairment)) OR (stroke or ‘cerebrovascular accident’) OR ((brain or cerebral or intracranial) adj2 (infarct* or thrombos?s or embolism)) OR ‘transient isc?emic attack’ OR tachycardi* OR ‘heart attack*’ OR (heart adj2 (attack or disease)) OR ‘left ventricular hypertrophy’ or cardiovascular disorders/ or exp heart disorders/ or exp arteriosclerosis/ or exp ischemia/ OR cerebrovascular disorders/ OR cerebral arteriosclerosis/ OR exp cerebral ischemia/ OR cerebrovascular accidents/	“cardiovascular disease*” OR ((coronary or isch?emic) adj2 (disease or occlusion or stenos#s or thrombos#s)) OR (myocardial adj (isch?emia or infarct*)) OR ((coronary or myocardial or heart or cardiac) adj2 (revasculari?ation or angioplasty or atherectomy or bypass)) OR ((heart or cardiac or ventricular) adj failure) OR angina OR ((ventricular or systolic or diastolic) adj (dysfunction or impairment)) OR (stroke or ‘cerebrovascular accident’) OR ((brain or cerebral or intracranial) adj2 (infarct* or thrombos?s or embolism)) OR ‘transient isc?emic attack’ OR tachycardi* OR ‘heart attack*’ OR (heart adj2 (attack or disease)) OR ‘left ventricular hypertrophy’ or cardiovascular disease/ OR ischemic heart disease/ OR exp acute coronary syndrome/ OR exp angina pectoris/ OR coronary artery atherosclerosis/ OR coronary artery obstruction/ OR coronary artery thrombosis/ OR exp heart infarction/ OR heart muscle ischemia/ OR ischemic cardiomyopathy/ OR angina pectoris/ OR heart muscle revascularization/ OR heart failure/ OR heart ventricle function/ cerebrovascular disease/ OR exp brain infarction/ OR exp brain ischemia/ OR exp cerebrovascular accident/	"cardiovascular disease*" OR ((coronary or isch#emic) N2 (disease or occlusion or stenos#s or thrombos#s)) OR (myocardial N (isch#emia or infarct*)) OR ((coronary or myocardial or heart or cardiac) N2 (revasculari#ation or angioplasty or atherectomy or bypass)) OR ((heart or cardiac or ventricular) N failure) OR angina OR ((ventricular or systolic or diastolic) N (dysfunction or impairment)) OR (stroke or "cerebrovascular accident") OR ("brain" OR "cerebral" OR "intracranial") W2 ("infarct*" OR "thrombos?s" OR "embolism") OR AB ("brain" OR "cerebral" OR "intracranial") W2 ("infarct*" OR "thrombos?s" OR "embolism") OR Tl ("ventricular" OR "systolic" OR "diastolic") W1 ("dysfunction" OR "impairment") OR AB ("ventricular" OR "systolic" OR "diastolic") W1 ("dysfunction" OR "impairment") OR Tl ("heart" OR "cardiac" OR "ventricular") W1 "failure" OR AB ("heart" OR "cardiac" OR "ventricular") W1 "failure" OR Tl ("coronary" OR "myocardial" OR "heart" OR "cardiac") W2 ("revasculari#ation" OR "angioplasty" OR "atherectomy" OR "bypass") OR AB ("coronary" OR "myocardial" OR "heart" OR "cardiac") W2 ("revasculari#ation" OR "angioplasty" OR "atherectomy" OR "bypass") OR "angina" OR (MH "Cerebral lschemia+") OR (MH "lntracranial Arterial Diseases+") OR (MH "lntracranial Embolism and Thrombosis+") OR (MH "Stroke+") OR (MH "Ventricular Dysfunction+") OR (MH "Heart Failure+") OR (MH "Myocardial Revascularization+") OR (MH "Myocardial lschemia+") OR (MH "Cardiovascular Diseases")	Cardiovascular diseases or ischemic heart diseases or acute coronary syndrome or myocardial ischemia or myocardial infarct or cardiac failure or ventricular dysfunction or angina or coronary artery atherosclerosis or coronary artery obstruction or thrombosis or heart infarct or ischemia or cardiomyopathy or stroke or cerebrovascular disease or heart failure

### Study selection

Studies will be deduplicated in Endnote and uploaded to Rayyan for screening. Two reviewers will independently undertake the screening process in two rounds: the first round will involve title and abstract screening, and the second round will focus on full-text screening. A third reviewer will resolve any discrepancies between the reviewers at each stage.

### Data extraction

Two reviewers will extract data independently according to a pre-piloted Microsoft Excel form. Discrepancies will be identified and resolved through discussion (with a third reviewer where necessary). Where study data is not reported ( *e.g.*, conference abstracts), authors will be contacted up to two times within a one-month period to request relevant data.

The extracted information will include authors, title, publication year, country, study design, data collection timeframe, setting, recruitment, inclusion/exclusion criteria, the number of participants, age, sex, ethnicity, psychosis diagnosis type, method of diagnosing, medication status, year of follow-up, method of diagnosing physical outcomes, prevalence/incidence estimates at baseline and at >0–12 months, or 13–36 months, or 37–60 months, or after more than 5 years after a FEP diagnosis, percentage smoking, comorbid mental disorders/substance abuse disorders, covariates adjusted for, control group/comparator data, funding sources/conflicts of interest, and additional information. In the case of missing data, study investigators will be contacted for unreported data or additional details. Selected articles will be stored and managed using EndNote X9 Reference Manager Library.

### Risk of bias in individual studies

Two reviewers will independently assess the risk of bias in included studies using the JBI Critical Appraisal Checklist for Studies Reporting Prevalence Data ( Munn *et al.*, 2015). Disagreements between reviewers will be resolved by discussion with a third reviewer.

### Strategy of data synthesis

A narrative synthesis will be used to synthesise and summarise the findings and to explore relationships in the data. If two or more studies are available, a random-effects meta-analysis of prevalence with 95% confidence and prediction intervals will be calculated for studies investigating outcomes within the same disease category (metabolic, cardiovascular, cancer or respiratory outcomes) at >0–12 months, at 13–36 months, at 37–60 months, and after more than 5 years after a FEP diagnosis ( *e.g.*, one meta-analysis on FEP cohort studies at 13–36 month follow up reporting the prevalence of respiratory outcomes). We will also conduct meta-analyses on the prevalence of specific physical health outcomes that are reported on in two or more studies at 0–12 months, at 13–36 months, at 37–60 months, and after more than 5 years.

Heterogeneity between prevalence estimates will be assessed using the I ^2^ statistic, Tau-squared, and prediction intervals. We will also generate Forest plots for the prevalence estimates and their 95% CI of the individual studies and pooled estimates. Forest plots will be examined visually, looking for potential outliers. We will conduct sensitivity analyses based on study quality and exclude studies at high risk of bias. Publication bias will be assessed with a funnel plot and the Begg and Egger tests.

### Analysis of subgroups

Sensitivity analyses will be conducted for associations supported by previous literature or convincing evidence. The following factors will be considered: study design, study year, studies that adjusted for age/sex, studies that adjusted for smoking at baseline, studies that adjusted for BMI or obesity at baseline, studies that adjusted for physical activity levels at baseline; studies that adjusted for the presence of co-occurring mental disorders at baseline; studies that adjusted for exposure to childhood maltreatment at baseline; studies that adjusted for use of psychotropic medications/substances; studies sponsored by a pharmaceutical company, and studies that adjusted for general medical burden at baseline.

A meta-regression analysis of moderators of interest will be conducted if there are more than 10 studies available on one specific illness ( *e.g.*, Metabolic syndrome) at one of the pre-specified time periods:

(a) Mean age

(b) Ethnicity

(c) Sex

(d) % smokers

(e) % taking antipsychotic medication

(f) % alcohol or substance use disorder

(g) Study year

### Public and patient involvement and dissemination plans

Public and Patient Involvement (PPI) has been actively engaged in the development of the research question and the systematic review protocol to ensure the relevance and appropriateness of this review. The completed review will be submitted to a leading journal in this field. Drawing from the insights of the review, we plan to create a recovery education module, informed by both the study findings and PPI feedback, to further support and inform patient groups. To make our findings accessible and comprehensible to a broader audience, a lay summary will be added to the review and disseminated to interested patient groups, with the continuous involvement of PPI to ensure its clarity and relevance.

## Study status

The systematic review is currently at the stage of data extraction. Database searching and screening have been completed.

## Discussion

Individuals with FEP experience significant health disparities, demonstrated by increased rates of premature mortality and a variety of physical comorbidities ( Firth *et al*., 2019). This systematic review seeks to consolidate the existing knowledge on the prevalence of physical health comorbidities among this group.

An important focus of our review is the health trajectory from FEP onset to subsequent stages. Past reviews often group FEP with chronic psychosis, potentially masking the trajectory of physical health comorbidities ( Leucht *et al*., 2007; Oud & Meyboom-de Jong, 2009). Our review dissects the prevalence of cardiovascular, metabolic, cancer, and respiratory comorbidities across varying post-diagnosis durations to provide a detailed health outlook for this cohort. A key component of our review evaluates the influence of demographic variables on prevalence rates. Understanding these demographic nuances can aid in public service planning and preparedness, ensuring communities are better equipped to address these challenges. By only including studies with antipsychotic-naïve individuals with FEP, we aim to achieve a better appreciation of the baseline physical health changes after entering mental health services.

We also anticipate certain limitations in our review. The reported prevalence rates may not accurately represent the true prevalence of specific physical health disorders among individuals with FEP. Prior research has indicated that conditions like cancer tend to be diagnosed at more advanced stages compared to the general population ( Wootten *et al*., 2022). Additionally, diagnostic overshadowing could further lead to underreporting physical health issues ( Molloy *et al*., 2023; Shefer *et al*., 2014).

## Data Availability

All data underlying the results are available as part of the article and no additional source data are required. Figshare: PRISMA-P checklist for ‘The short-, medium-, and long-term prevalence of physical health comorbidities in first-episode psychosis: a systematic review protocol’. https://doi.org/10.6084/m9.figshare.24162756 ( Zierotin *et al.*, 2023). Data are available under the terms of the Creative Commons Attribution 4.0 International license (CC-BY 4.0).
