# Supplementary material for: The short-, medium-, and long-term prevalence of physical health comorbidities in first-episode psychosis: a systematic review and meta-analysis protocol
Source: HRB Open Res. 2025 Jan 8;6:75. Originally published 2023 Dec 7. [Version 2] doi: 10.12688/hrbopenres.13810.2 (PMC11757914; doi:10.12688/hrbopenres.13810.2)
Supplement: Peer review history

# Reviewer report: Reviewer response for version 2

**DOI:** 10.21956/hrbopenres.15441.r44661

**Published:** 2025-01-23

**Authors:** A Dregan

**Affiliations:** 1 King's College London, London, England, UK

I am satisfied with the authors responses to my earlier concerns and I have no further comments. I recommend the article for indexing.

Is the study design appropriate for the research question?

Yes

Is the rationale for, and objectives of, the study clearly described?

Partly

Are sufficient details of the methods provided to allow replication by others?

Partly

Are the datasets clearly presented in a useable and accessible format?

Not applicable

Reviewer Expertise:

NA

I confirm that I have read this submission and believe that I have an appropriate level of expertise to confirm that it is of an acceptable scientific standard.

# Reviewer report: Reviewer response for version 2

**DOI:** 10.21956/hrbopenres.15441.r44805

**Published:** 2025-01-23

**Authors:** Anna Waterreus

**Affiliations:** 1 University of Western Australia, Crawley, Australia

Thank you for asking me to review this revised study protocol for a systematic review and meta-analysis investigating the prevalence of physical health co-morbities over time in people in their first episode of psychosis.

 This document clearly outlines the rationale and methodology for this study. 

 The abstract and introduction states that people are followed from FEP onset, however, the description in the eligibility criteria is more accurate in that people are being followed from first diagnosis. In some countries the period of time between onset and diagnosis may be short but in others it may not be.

 In the last paragraph in Methods the sentence "There will be no restrictions regarding language or publication period." is repeated twice and needs correcting.

 Data extraction

 A supplementary file which includes the data extract form would enhance the study's replicability.

 Data with be extracted on co-morbid substance use disorders. However, what about those people who are using illicit substances but are not diagnosed with a disorder?

 Cannabis is commonly used by people with psychotic disorders and may have a positive impact on cardiometabolic risk factors in people with psychotic disorders, in particular in regard to obesity and diabetes, as shown in my 2016 paper. ^1 ^A Dutch study ^2^ of people with psychotic or mood disorders, reported that cannabis users had smaller waist circumferences and lower diastolic blood pressure than non-users, and Liemburg et al. ^3^ documented that cannabis use was associated with lower BMI in people with psychotic disorders. Studies ^4-6^ in first-episode psychosis participants showed cannabis users had a better metabolic profile than non-users and that, over time, cannabis users gained significantly less weight and were less likely to experience harmful changes in other metabolic measures.

^1^Waterreus A, et al. 2016 (Ref 1)

^2 ^Bruins J, et al 2016 (Ref 2)

^3 ^Liemburg EJ, et al. 2016 (Ref 3)

^4 ^Scheffler F, et al. 2018 (Ref 4)

^5 ^Vázquez-Bourgon J, et al. 2019 (Ref 5)

^6 ^Stiles E, et al. 2020 (Ref 6)

Is the study design appropriate for the research question?

Yes

Is the rationale for, and objectives of, the study clearly described?

Yes

Are sufficient details of the methods provided to allow replication by others?

Partly

Are the datasets clearly presented in a useable and accessible format?

Not applicable

Reviewer Expertise:

My research focuses on people with psychotic disorders and their physical health outcomes.

I confirm that I have read this submission and believe that I have an appropriate level of expertise to confirm that it is of an acceptable scientific standard.

## References

[1] : Metabolic syndrome in people with a psychotic illness: is cannabis protective?. *Psychol Med* .2016;46(8) : 10.1017/S0033291715002883 1651-62 10.1017/S0033291715002883 26965714

[2] : Cannabis use in people with severe mental illness: The association with physical and mental health--a cohort study. A Pharmacotherapy Monitoring and Outcome Survey study. *J Psychopharmacol* .2016;30(4) : 10.1177/0269881116631652 354-62 10.1177/0269881116631652 26883305

[3] : Cannabis and a lower BMI in psychosis: What is the role of AKT1?. *Schizophr Res* .2016;176(2-3) : 10.1016/j.schres.2016.08.014 95-99 10.1016/j.schres.2016.08.014 27554198

[4] : Effects of cannabis use on body mass, fasting glucose and lipids during the first 12 months of treatment in schizophrenia spectrum disorders. *Schizophr Res* .2018;199: 10.1016/j.schres.2018.02.050 90-95 10.1016/j.schres.2018.02.050 29519756

[5] : Effect of cannabis on weight and metabolism in first-episode non-affective psychosis: Results from a three-year longitudinal study. *J Psychopharmacol* .2019;33(3) : 10.1177/0269881118822173 284-294 10.1177/0269881118822173 30702972

[6] : Cannabis use and metabolic syndrome among clients with first episode psychosis. *Early Interv Psychiatry* .2021;15(4) : 10.1111/eip.13030 1051-1055 10.1111/eip.13030 32881419 PMC7921202

# Reviewer report: Reviewer response for version 2

**DOI:** 10.21956/hrbopenres.15441.r44660

**Published:** 2025-01-10

**Authors:** Luigi Francesco Saccaro

**Affiliations:** 1 University of Geneva, Geneva, Switzerland

The authors have addressed all comments appropriately, and I look forward to the results of this interesting meta-analysis. Best of luck!

Is the study design appropriate for the research question?

Partly

Is the rationale for, and objectives of, the study clearly described?

Yes

Are sufficient details of the methods provided to allow replication by others?

Partly

Are the datasets clearly presented in a useable and accessible format?

Not applicable

Reviewer Expertise:

Psychiatry

I confirm that I have read this submission and believe that I have an appropriate level of expertise to confirm that it is of an acceptable scientific standard.

# Reviewer report: Reviewer response for version 1

**DOI:** 10.21956/hrbopenres.15114.r43606

**Published:** 2024-12-10

**Authors:** Luigi Francesco Saccaro

**Affiliations:** 1 University of Geneva, Geneva, Switzerland

GENERAL COMMENTS

 This well-designed, preregistered protocol clearly outlines a systematic review and meta-analysis investigating the short-, medium-, and long-term prevalence of metabolic, cardiovascular, respiratory, and oncologic comorbidities in antipsychotic-naïve or minimally exposed individuals with FEP, aiming to understand the progression of physical health comorbidities.

 Observational longitudinal studies and antipsychotic intervention studies will primarily be reviewed to estimate the prevalence of comorbidities across different time intervals and to explore demographic influences. The protocol is expected to adhere to PRISMA and MOOSE guidelines, incorporating a comprehensive search strategy across multiple databases and grey literature, dual independent screening, data extraction, and risk of bias assessment using validated tools (e.g., the JBI Critical Appraisal Checklist). The planned meta-analyses of prevalence data, along with sensitivity analyses and meta-regressions, appear methodologically sound.

 Finally, the study intends to generate evidence applicable to early intervention strategies, with a focus on incorporating public and patient involvement (PPI) for dissemination and practical application.

 While the clinical relevance and interest of the review are clear, several points need to be addressed, as outlined below.

 TITLE

 The title is appropriate, but I suggest including a reference to the potential meta-analysis.

 ABSTRACT

 Please briefly mention the main expected limitations in the abstract.

 INTRODUCTION

 The introduction is well-written and provides appropriate context.

 METHODS

 The protocol should specify the cutoff sample size to define and exclude a case series, for example, n<11, to ensure clarity in eligibility criteria.

 While the protocol mentions contacting authors twice for missing data, it does not outline fallback strategies for unresponsive authors (a common situation unfortunately…). Alternative approaches, such as imputing missing data or excluding studies, should be provided to address this limitation.

 A supplementary table summarizing inclusion and exclusion criteria in a structured format (e.g., PICOS or CoCoPop) would enhance transparency. This table should include a detailed list of DSM-5 diagnoses under consideration. For example, it is unclear whether Type I bipolar disorder is included, and a justification for either choice is necessary.

 The exclusion of personality disorders should be better justified given the increasing body of literature exploring the psychosis spectrum.

 Additionally, the protocol should provide further details on how studies involving overlapping populations will be identified and managed to avoid duplication and ensure accurate synthesis.

 More specific details on the involvement of patients and the public (PPI) in the design of the protocol would be helpful to clarify the extent of their contributions.

 The protocol should also include plans to extract data on average chlorpromazine equivalents or another standardized measure of antipsychotic dosage as a covariate, to facilitate comparisons across studies.

 There should be greater specificity regarding how inconsistencies in diagnostic criteria between studies, such as differences between ICD-10 and ICD-11, will be harmonized or accounted for in the analysis.

 Finally, the thresholds of 5% for antipsychotic exposure and 20% for neurodivergence require justification, ideally supported by prior literature or pilot analyses, to ensure these decisions are evidence-based.

 DISCUSSION & CONCLUSIONS

 The discussion should expand on the limitations of the study, particularly those highlighted in the methods section that could not be fully addressed. For instance, the implications of excluding personality disorders and the potential impact of diagnostic inconsistencies on the findings should be explicitly discussed.

 The representativeness of the included studies is another area requiring attention. For example, studies from high-income countries may not reflect the prevalence or progression of physical health comorbidities in low- and middle-income settings. This disparity should be explicitly acknowledged, and its implications for generalizability should be considered.

 Further details on the concrete clinical implications of the review's findings would strengthen the discussion. For example, how might the findings inform potential interventions or changes in clinical practice? Suggesting future research directions, such as investigating the mechanisms underlying disparities in physical health outcomes or conducting longitudinal studies to evaluate intervention effectiveness, would also add value.

 MINOR COMMENTS

 All abbreviations should be defined upon their first use, including those in table legends. Abbreviations should then be used consistently throughout the text. For example, “FEP” and “first-episode psychosis” are used interchangeably in the current draft, and this inconsistency should be corrected.

 Additionally, typos and grammatical mistakes should be corrected to improve the overall readability of the manuscript.

Is the study design appropriate for the research question?

Partly

Is the rationale for, and objectives of, the study clearly described?

Yes

Are sufficient details of the methods provided to allow replication by others?

Partly

Are the datasets clearly presented in a useable and accessible format?

Not applicable

Reviewer Expertise:

Psychiatry

I confirm that I have read this submission and believe that I have an appropriate level of expertise to confirm that it is of an acceptable scientific standard, however I have significant reservations, as outlined above.

# Response

**Published:** 2025-01-02

**Authors:** Anna Zierotin

**Affiliations:** University College Dublin, Dublin, Ireland

1. TITLE

 The title is appropriate, but I suggest including a reference to the potential meta-analysis.

*We thank the Reviewer for their comment. We have revised the manuscript title:*

 Page 1

 “The short-, medium-, and long-term prevalence of physical health comorbidities in first-episode psychosis: a systematic review and meta-analysis protocol.”

 2. ABSTRACT

 Please briefly mention the main expected limitations in the abstract.

***Response:** We thank the Reviewer for their comment. We have added a sentence in the Abstract highlighting potential limitations, including heterogeneity across studies in diagnostic criteria and the risk of underreporting certain physical comorbidities.*

 Page 3

 “Potential limitations include the risk of diagnostic heterogeneity across studies and possible underreporting of certain comorbidities.”

 3. INTRODUCTION

 The introduction is well-written and provides appropriate context.

 4. METHODS

 The protocol should specify the cutoff sample size to define and exclude a case series, for example, n<11, to ensure clarity in eligibility criteria.

***Response: **We thank the Reviewer for their comment. In the revised manuscript, we now specify that studies with fewer than 10 participants will be classified as case series and excluded. This numeric cutoff (n<10) is stated to ensure clarity in our eligibility criteria.*

 Page 5

 “We will exclude single case studies and case series, specifically defining studies with fewer than 10 participants as case series.”

 5. While the protocol mentions contacting authors twice for missing data, it does not outline fallback strategies for unresponsive authors (a common situation unfortunately…). Alternative approaches, such as imputing missing data or excluding studies, should be provided to address this limitation.

***Response: **We thank the Reviewer for their comment.* *We have updated the manuscript to clarify our approach for unresponsive authors: if authors do not reply after two contact attempts within one month, we will proceed without the missing data and consider using sensitivity analyses or excluding that study if the missing data critically affect results.*

 Page 15

 “If authors remain unresponsive, we will either proceed with analyses excluding the missing data or, if the absence critically impairs the study’s utility, exclude that study from meta-analyses and note it in sensitivity analyses. We will also collect data on average chlorpromazine equivalents or another standardized antipsychotic dosage measure when reported, to facilitate cross-study comparisons.”

 6. A supplementary table summarizing inclusion and exclusion criteria in a structured format (e.g., PICOS or CoCoPop) would enhance transparency. This table should include a detailed list of DSM-5 diagnoses under consideration. For example, it is unclear whether Type I bipolar disorder is included, and a justification for either choice is necessary.

***Response: **We thank the Reviewer for their comment.* *We have added a new supplementary Table S1 in the revised protocol, presenting our inclusion/exclusion criteria.*

 7. The exclusion of personality disorders should be better justified given the increasing body of literature exploring the psychosis spectrum.

***Response: **We thank the Reviewer for their comment. After re-evaluating our approach, we have removed the strict exclusion of personality disorders from our protocol. We will instead include all studies that meet our definition of first-episode psychosis (FEP), acknowledging that a subset of participants may have co-occurring personality disorders.*

 8. Additionally, the protocol should provide further details on how studies involving overlapping populations will be identified and managed to avoid duplication and ensure accurate synthesis.

***Response: **We thank the Reviewer for their comment. We have included the below statement that we will examine study IDs, recruitment sites, time frames, and researcher groups to identify overlapping populations. If we identify multiple reports from the same cohort, we will include the most comprehensive dataset.*

 Page 15

 “We will check for overlapping cohorts by examining recruitment site, time frame, and researcher groups. If multiple publications arise from the same study population, we will include the most comprehensive data set.”

 9. More specific details on the involvement of patients and the public (PPI) in the design of the protocol would be helpful to clarify the extent of their contributions.

***Response: **We thank the reviewer for their comment. We included more detail on the involvement of PPI in the design of the protocol.*

 Page 17

 “One of the co-authors has lived experience of FEP and serves as an expert by experience, having actively contributed to formulating the protocol and shaping the research question. They will also be involved in interpreting the findings and co-creating dissemination materials.”

 10. The protocol should also include plans to extract data on average chlorpromazine equivalents or another standardized measure of antipsychotic dosage as a covariate, to facilitate comparisons across studies.

***Response: **We thank the Reviewer for their comment. We have updated our data extraction plan to include collection of chlorpromazine-equivalent dosages or similar standardized measures when reported.*

 Page 15

 “We will also collect data on average chlorpromazine equivalents or another standardized antipsychotic dosage measure when reported, to facilitate cross-study comparisons.”

 11. There should be greater specificity regarding how inconsistencies in diagnostic criteria between studies, such as differences between ICD-10 and ICD-11, will be harmonized or accounted for in the analysis.

***Response: **We thank the Reviewer for their comment. We included an explanation that all reported diagnoses will first be recorded as stated (e.g., ICD-10, ICD-11), and where necessary, we will conduct subgroup or sensitivity analyses to account for diagnostic discrepancies. We will also cross-reference ICD-11 criteria when outcomes are ambiguously classified.*

 12. Finally, the thresholds of 5% for antipsychotic exposure and 20% for neurodivergence require justification, ideally supported by prior literature or pilot analyses, to ensure these decisions are evidence-based.

***Response: **We thank the Reviewer for their comment. After reconsidering the feedback, we have decided to remove the strict exclusion criteria and associated thresholds for organic psychotic disorders, personality disorders, and neurodevelopmental disorders. Instead, we will include all studies where participants meet a first-episode psychosis (FEP) definition, without imposing a predefined maximum percentage of comorbid presentations, to better capture real-world clinical populations.* *Regarding antipsychotic exposure, we will maintain the 28-day principle as a guideline for identifying ‘antipsychotic-naïve or minimally exposed’ cohorts, but we will not exclude studies solely if a small proportion of participants exceed this duration. Rather than enforcing a strict 5% threshold, we will record each study’s criteria for antipsychotic exposure, note the proportion of participants exceeding 28 days, and where data permit, conduct sensitivity analyses to determine whether including those with slightly extended exposure materially alters our results. This approach preserves the intent of capturing truly early-stage cohorts while recognizing practical variations in clinical settings.*

 Page 6

 “If a study includes a subset with longer exposure, we will not automatically exclude it but will document the proportion and, if feasible, conduct sensitivity analyses to examine any impact on overall findings.”

 13. DISCUSSION & CONCLUSIONS

 The discussion should expand on the limitations of the study, particularly those highlighted in the methods section that could not be fully addressed. For instance, the implications of excluding personality disorders and the potential impact of diagnostic inconsistencies on the findings should be explicitly discussed.

 The representativeness of the included studies is another area requiring attention. For example, studies from high-income countries may not reflect the prevalence or progression of physical health comorbidities in low- and middle-income settings. This disparity should be explicitly acknowledged, and its implications for generalizability should be considered.

***Response: **We thank the reviewer for their comment. We have revised the Discussion to more explicitly acknowledge these limitations, explaining how our strict inclusion/exclusion criteria may affect generalizability. We also emphasize possible geographical bias, noting that published studies on FEP often come from high-income nations, potentially limiting applicability to low-resource settings.*

 Page 17

 “We acknowledge that excluding personality disorders may limit the generalizability of our findings to individuals on the broader psychosis spectrum. Additionally, diagnostic differences (e.g., ICD-10 vs. ICD-11) and variation in how outcomes are measured may introduce heterogeneity in prevalence estimates, which we aim to address via subgroup or sensitivity analyses. We also note that many included studies are likely to be conducted in high-income countries, which can reduce the representativeness of the results in low- and middle-income contexts. Despite these potential limitations, the findings of this review will provide insights that can inform clinical practice and early intervention strategies”

 14. Further details on the concrete clinical implications of the review's findings would strengthen the discussion. For example, how might the findings inform potential interventions or changes in clinical practice? Suggesting future research directions, such as investigating the mechanisms underlying disparities in physical health outcomes or conducting longitudinal studies to evaluate intervention effectiveness, would also add value.

***Response: **We thank the Reviewer for their comment. We included more details on the concrete clinical implications and future research directions in the discussion.*

 Page 18

 “The findings can inform targeted interventions and timely management of metabolic, cardiovascular, respiratory, and oncologic risks, as well as highlight the need for embedding structured physical health assessments and integrated care strategies into early intervention services for FEP, informing. Future longitudinal research should evaluate the effectiveness of these approaches while examining genetic, lifestyle, and socioeconomic mechanisms underlying disparities in physical health outcomes.”

 15. MINOR COMMENTS

 All abbreviations should be defined upon their first use, including those in table legends. Abbreviations should then be used consistently throughout the text. For example, “FEP” and “first-episode psychosis” are used interchangeably in the current draft, and this inconsistency should be corrected.

 Additionally, typos and grammatical mistakes should be corrected to improve the overall readability of the manuscript.

***Response: **We thank the Reviewer for their comment. We have carefully reviewed the manuscript to ensure each abbreviation (FEP) is defined at first mention and used consistently thereafter. We have also checked the manuscript for typographical and grammatical errors.*

# Reviewer report: Reviewer response for version 1

**DOI:** 10.21956/hrbopenres.15114.r38110

**Published:** 2024-03-14

**Authors:** A Dregan

**Affiliations:** 1 King's College London, London, England, UK

The work represents the protocol of a proposed study to synthesize current evidence on the link between FEP and antipsychotic drugs with a restricted number of major physical long-term conditions.The questions are well detailed and clear, and the search strategy and data resources look appropriate for the purposes of the review. This Reviewer has several major questions that needs clarification. 

 1. Why not focusing on the bidirectional association between FEP and physical LTCs? Some young people with FEP will have pre-existing LTCs that might be negatively impacted by antipsychotic prescribing/FEP symptoms. This is particularly relevant given the focus on prevalence rather than incidence of physical LTCs.

 2.  Antipsychotics are not specific to FEP ( severe anxiety, mood) - would the review exclude interventions in non-FEP patients? 

 3. Related to 2. - antipsychotics might moderate/mediate the association between FEP and physical LTCs - should be included as a specific aim/RQ. 

 4. Some of the details in the Population section might be best placed in the Eligibility criteria and further justified. For instance, why the need to exclude organic psychotic disorders? 

 5. How would quality of included studies be assessed?

 6. Social deprivation is a major determinant of FEP and physical LTCs, as it is parental mental health history. Might be worth extending the moderators list.

Is the study design appropriate for the research question?

Yes

Is the rationale for, and objectives of, the study clearly described?

Partly

Are sufficient details of the methods provided to allow replication by others?

Partly

Are the datasets clearly presented in a useable and accessible format?

Not applicable

Reviewer Expertise:

Multiple long-term conditions epidemiology and patterning in people with mental health conditions.

I confirm that I have read this submission and believe that I have an appropriate level of expertise to confirm that it is of an acceptable scientific standard, however I have significant reservations, as outlined above.

# Response

**Published:** 2025-01-02

**Authors:** Anna Zierotin

**Affiliations:** University College Dublin, Dublin, Ireland

The work represents the protocol of a proposed study to synthesize current evidence on the link between FEP and antipsychotic drugs with a restricted number of major physical long-term conditions. The questions are well detailed and clear, and the search strategy and data resources look appropriate for the purposes of the review. This Reviewer has several major questions that needs clarification. 

 1. Why not focusing on the bidirectional association between FEP and physical LTCs? Some young people with FEP will have pre-existing LTCs that might be negatively impacted by antipsychotic prescribing/FEP symptoms. This is particularly relevant given the focus on prevalence rather than incidence of physical LTCs.

*We thank the Reviewer for their comment. In the revised protocol, we now acknowledge that some individuals may have pre-existing LTCs prior to, or at the time of, their FEP diagnosis. We have added a clarifying statement in the Introduction and Methods sections to note that while our primary focus is on the short-, medium-, and long-term prevalence of specific LTCs post-FEP diagnosis, we recognize that some comorbid conditions might precede FEP or be exacerbated by antipsychotic treatment and have added it to our data extraction.*

 Page 4

 “ It also considers that certain comorbid conditions may have been present prior to FEP diagnosis and can be exacerbated by FEP or subsequent antipsychotic treatment.”

 Page 5

 “Since some participants may have pre-existing comorbidities prior to FEP, the data extraction will consider whether these conditions emerged prior to or following FEP diagnosis.”

 2.  Antipsychotics are not specific to FEP ( severe anxiety, mood) - would the review exclude interventions in non-FEP patients? 

*We thank the Reviewer for their comment. Our review aims to include only those antipsychotic intervention studies where participants are antipsychotic-naïve FEP individuals or have no more than 28 days’ antipsychotic exposure (as per Maudsley clinical guidelines). We have clarified in the Eligibility Criteria section that studies focusing on non-FEP populations (e.g., those with severe anxiety or mood disorders alone) will be excluded.*

 Page 5

 “Studies that primarily focus on non-FEP populations, such as those with severe anxiety or mood disorders will be excluded. If mixed samples (for example, severe mental illness groups) are used, we will attempt to extract the data specific to people with psychosis.”

 3. Related to 2. - antipsychotics might moderate/mediate the association between FEP and physical LTCs - should be included as a specific aim/RQ. 

*We thank the Reviewer for their comment. We included the role of antipsychotic medication as a potential moderating or mediating factor between FEP and physical comorbidities as a specific research question:*

 Page 5

 “2. To what extent do antipsychotic medications moderate or mediate the association between FEP and these physical comorbidities?”

 4. Some of the details in the Population section might be best placed in the Eligibility criteria and further justified. For instance, why the need to exclude organic psychotic disorders? 

*We thank the Reviewer for their comment. We have removed the blanket exclusion of organic psychotic disorders from the revised protocol to ensure that our review reflects the heterogeneity of first-episode psychosis presentations.*

 5. How would quality of included studies be assessed?

*We thank the Reviewer for their comment. We will use the JBI Critical Appraisal Checklist for Studies Reporting Prevalence Data to assess the quality of included studies:*

 Page 15

 “Two reviewers will independently assess the risk of bias in included studies using the JBI Critical Appraisal Checklist for Studies Reporting Prevalence Data ( Munn et al., 2015). Disagreements between reviewers will be resolved by discussion with a third reviewer. This tool will help assess study quality based on criteria such as sample representativeness, reliability of outcome measurement, and appropriateness of statistical analysis. The results of this appraisal will inform sensitivity analyses, where we may exclude high-risk studies to assess their impact on pooled estimates.”

 6. Social deprivation is a major determinant of FEP and physical LTCs, as it is parental mental health history. Might be worth extending the moderators list.

*We thank the Reviewer for their comment. We have now extended the Analysis of subgroups section to include social deprivation status as well as parental mental health history where reported. We note that these factors will be extracted from studies if provided, and we will conduct subgroup/meta-regression analyses on them where there are sufficient data (≥10 studies).*

 Page 15

 “The extracted information will include authors, title, publication year, country, study design, data collection timeframe, setting, recruitment, inclusion/exclusion criteria, the number of participants, age, sex, ethnicity, psychosis diagnosis type, method of diagnosing, medication status, year of follow-up, method of diagnosing physical outcomes, prevalence/incidence estimates at baseline and at >0–12 months, or 13–36 months, or 37–60 months, or after more than 5 years after a FEP diagnosis, percentage smoking, comorbid mental disorders/substance abuse disorders, social deprivation measures, parental mental health history, covariates adjusted for, control group/comparator data, funding sources/conflicts of interest, and additional information.”

 Page 16

 “Analysis of Subgroups

 …We will also include additional variables such as social deprivation status and parental mental health history if reported.”
